# Liver X Receptor Regulation of Thyrotropin-Releasing Hormone Transcription in Mouse Hypothalamus Is Dependent on Thyroid Status

**DOI:** 10.1371/journal.pone.0106983

**Published:** 2014-09-17

**Authors:** Rym Ghaddab-Zroud, Isabelle Seugnet, Knut R. Steffensen, Barbara A. Demeneix, Marie-Stéphanie Clerget-Froidevaux

**Affiliations:** 1 CNRS UMR 7221-USM 501 « Evolution of Endocrine Regulations », « Regulations, Development and Molecular Diversity » department, Muséum National d’Histoire Naturelle, CP32, Paris, France; 2 Division of Clinical Chemistry, Department of Laboratory Medicine, Karolinska Institutet, Karolinska University Hospital Huddinge, Stockholm, Sweden; INRA, France

## Abstract

Reversing the escalating rate of obesity requires increased knowledge of the molecular mechanisms controlling energy balance. Liver X receptors (LXRs) and thyroid hormone receptors (TRs) are key physiological regulators of energetic metabolism. Analysing interactions between these receptors in the periphery has led to a better understanding of the mechanisms involved in metabolic diseases. However, no data is available on such interactions in the brain. We tested the hypothesis that hypothalamic LXR/TR interactions could co-regulate signalling pathways involved in the central regulation of metabolism. Using *in vivo* gene transfer we show that LXR activation by its synthetic agonist GW3965 represses the transcriptional activity of two key metabolic genes, Thyrotropin-releasing hormone (*Trh)* and Melanocortin receptor type 4 (*Mc4r*) in the hypothalamus of euthyroid mice. Interestingly, this repression did not occur in hypothyroid mice but was restored in the case of *Trh* by thyroid hormone (TH) treatment, highlighting the role of the triiodothyronine (T_3_) and TRs in this dialogue. Using shLXR to knock-down LXRs *in vivo* in euthyroid newborn mice, not only abrogated *Trh* repression but actually increased *Trh* transcription, revealing a potential inhibitory effect of LXR on the Hypothalamic-Pituitary-Thyroid axis. *In vivo* chromatin immunoprecipitation (ChIP) revealed LXR to be present on the *Trh* promoter region in the presence of T_3_ and that Retinoid X Receptor (RXR), a heterodimerization partner for both TR and LXR, was never recruited simultaneously with LXR. Interactions between the TR and LXR pathways were confirmed by qPCR experiments. T_3_ treatment of newborn mice induced hypothalamic expression of certain key LXR target genes implicated in metabolism and inflammation. Taken together the results indicate that the crosstalk between LXR and TR signalling in the hypothalamus centres on metabolic and inflammatory pathways.

## Introduction

Obesity contributes to the aetiology of common associated metabolic diseases [1]–[3]. The causes of obesity are multifactorial, with environmental and genetic components. Westernized lifestyle, with high-caloric diets and a lack of physical exercise, is an obvious factor of obesity [4], but obesity does not develop in all individuals exposed to an obesogenic environment. Such observations emphasize the need to increase our knowledge of the molecular pathways and mechanisms involved in controlling energy balance.

In the periphery, regulation of energetic metabolism involves several types of nuclear receptors (NR). Among NRs, LXRs, activated by cholesterol metabolites, are known to be key regulators of lipid and cholesterol metabolism. The two related LXRs, LXRα (NR1H3) and LXRβ (NR1H2) are part of the emerging significant newer drug targets within the NR family (for review see [5]). A second type of NR, TRs, plays a major role in controlling energy metabolism. THs are known to regulate, at a transcriptional level, all the steps of cholesterol metabolism, and TRβ1 is the main receptor isoform involved [6]. Given the role played by these NR in metabolism, dysregulations of metabolic functions controlled by LXR/TRs can alter the homeostatic control circuits, contributing to the pathogenesis of many common metabolic diseases, such as obesity, insulin resistance, type 2 diabetes, hyperlipidaemia, atherosclerosis, and gallbladder disease [7]. Interactions between LXR and TR have been described in the periphery [8], [9]. These interactions are based on two major points: First, LXRs and TRs both use retinoid X receptors (RXRs) as common heterodimerization partner [10], a fact that can engender competition for RXR if quantities are limiting. Second, the consensus response elements (RE) for LXRs and TRs are very similar (basically a DR4). However, so far no data are available on how such interactions affect regulations at the central level, whereas, it is well known that hypothalamus is considered as the central integrator of metabolic regulation (for review see [11]). Consequently, the key players are the central controls of food intake, relayed by neural and gene networks in different hypothalamic nuclei. Indeed, in the context of thyroid hormone (TH)- induced gene regulation, our group has focused on hypothalamic interactions between different signalling pathways controlling metabolism [12], [13]. In particular, we showed that TH, through TRs, is directly involved in transcriptional regulation of melanocortin receptor type 4 (*Mc4r*) [12]. Moreover, the need for a detailed study on the involvement of LXR in central metabolic pathways and control of energy homeostasis is underlined by the fact that it has recently been shown that the central melanocortin pathway, particularly hypothalamic MC4R is involved in the control of hepatic cholesterol metabolism: in addition to facilitating hepatic cholesterol synthesis, the central melanocortin system influences cholesterol transport by modulating HDL cholesterol levels [14]. These results lead to the hypothesis that LXR signalling, in the hypothalamus, may interact with this pathway.

In the current study, we analysed, at the hypothalamic level, how LXR may interfere with transcriptional regulation induced by TRs. Using *in vivo* gene transfer we show that activation of LXR by its synthetic agonist GW3965 represses the transcriptional activity of two known TH target genes involved in the central control of metabolism, *Trh* and *Mc4r* promoters, and this only in euthyroid mice. This repression was restored by TH treatment in hypothyroid mice. By *in vivo* ChIP, we showed that LXR is recruited to the *Trh* promoter region in the presence of T_3_ but not in its absence. In contrast, RXR is recruited to the same region in the absence of T_3_. There is no simultaneous presence of RXR and LXR on *Trh* promoter region, suggesting that the presence of a receptor excludes the other. The interactions between the two pathways were further confirmed by qPCR, showing that T_3_ treatment of newborn mice induced hypothalamic regulation of a number of key LXR target genes implicated in metabolism and inflammation.

Thus, our data provide insight into molecular pathways involved in central metabolic regulation, showing for the first time a central crosstalk between TR and LXR pathways.

## Materials and Methods

### Ethics statement

Animal care and experimentation were in accordance with the National Institutes of Health Guidelines for the Care and Use of Laboratory Animals and approved by the Museum National d’Histoire Naturelle Animal Care and Use Committee, Paris, France.

### Experimental protocol

#### Animals

Swiss wild-type mice were from Janvier (Le Genest St Isle, France).

#### Hypothyroidism

To induce fetal and neonatal hypothyroidism, dams were given iodine-deficient food containing 0.15% 6-*n*-propyl-2-thiouracil (PTU) (Harlan, Gannat, France) from day 14 of gestation and through lactation.

#### T_3_ treatment

To assess T_3_ (Sigma-Aldrich) effect on restoring ligand-dependent repression of the promoters of *Trh* and *Mc4r* in the hypothalamus via activation of LXR by GW3965, hypothyroid pups were injected subcutaneously (s.c.) with 2.5 µg/g body weight (bw) of T_3_ in 0.9% saline 24 hours before transfection. Pups were decapitated 24 h after transfection and hypothalami were dissected for luciferase assays following the manufacturer’s protocol (Promega).

In the qPCR experiments assessing response to T_3_, we used a s.c. T_3_ (2.5 µg/g bw) or vehicle (0.9% saline) injection 6 h and 18 h before sacrifice and dissection.

#### Thyroxine (T_4_) treatment

Dams were treated with 1.2 µg/ml of T_4_ in drinking water from 24 or 48 hours before giving birth and the treatment in the drinking water was continued through lactation.

### Plasmids

TRH-Firefly luciferase (TRH-f.luc) was from PlasmidFactory (manufacturing code: pF312) and contains −547 to +84 bp of the TRH promoter cloned upstream of the Firefly luciferase-coding sequence [15]. MC4R-*Renilla* luciferase (MC4R-r.luc) was already published [12]. To knock-down endogenous LXRs, shRNA-coding plasmids against LXRα (pCMV-H1- shLXRα) and against LXRβ (pCMV-H1- shLXRβ) were designed according to validated shRNA sequences published in [16] and cloned into CMV-H1 as already described [12]. Each shRNA-coding sequence was purchased from MWG Eurofins. The control plasmid used was empty CMV-H1 [12]. For shLXRα, the sequence is: CTC GAG TGC CTG ATG TTT CTC CTG ATT CAA GAG ATC AGG AGA AAC ATC AGG CAT TGC GGC CGC; For shLXRβ, the sequences is: CTC GAG GAT TCA GAA GCA GCA ACA TTC AAG AGA TGT TGC TGC TTC TGA ATC CTT GCG GCC GC.

### Reagents

LXR agonist GW3965 (3-[3-[N-(2-Chloro-3-trifluoromethylbenzyl)-(2,2-diphenylethyl) amino] propyloxy] phenylacetic acid hydrochloride) was purchased from (Sigma–Aldrich) and dissolved in 100% dimethyl sulfoxide (DMSO) (Sigma–Aldrich) as a 50 mM stock solution at −20°C.

### In vivo transfection and luciferase assays

DNA/PEI complexes and *in vivo* gene transfer were adapted from [17]. Pups were anesthetized by hypothermia on ice and transfected on postnatal day 1 (in absence of T_3_ treatment or when GW3965 is added in the transfection mix) or day 2 (when T_3_ or GW3965 subcutaneous treatment is done on day 1). A glass micropipette was lowered 2.5 mm through the skull, 0.5 mm posterior to bregma and 0.5 mm lateral to the sagittal suture, into the hypothalamic area (paraventricular nucleus, PVN). Two microliters of a 5% glucose solution containing plasmid/polyethylenimine (PEI) complexes were slowly injected bilaterally into the hypothalamus.

For transfection of both MC4R and TRH reporter plasmids, newborn mice were cotransfected bilaterally in the hypothalamic region of the brain with 4 µL (2×2 µl) of a 250 ng/µl solution of MC4R-r.luc (0.8 µg/pup) and TRH-f.luc (0.2 µg/pup), complexed with PEI. For shLXR experiments, one-day-old euthyroid pups were transfected with 4 µl of a solution of a transfection mix containing PEI-complexed TRH-f.luc (0.2 µg/pup)/empty CMV-H1 (0.4 µg/pup) or a mix of CMV-H1shLXRα (0.2 µg/pup) and CMV-H1shLXRβ (0.2 µg/pup) with or without GW3965 at 10^−6^ M.

To assess the pharmacological activation of endogenous LXR, GW3965 diluted stock solution was added to the plasmids/PEI complex solution to reach final concentrations (10^−7^ or 10^−6^ M). In control animals, diluted DMSO was added to the plasmids/PEI complex solution.

Renilla and Firefly luciferase activities were measured 24 h later on each dissected hypothalamic region.

### G3965 treatment

To assess the pharmacological activation of endogenous LXR, GW3965 was either added to the transfection mix (10^−7^ or 10^−6^ M), or subcutaneously injected at 12.5 mg/Kg or 25 mg/Kg of body weight 24 hours before the transfection.

### In vivo chromatin immunoprecipitation

Pups were treated with T_3_ or vehicle and sacrificed 20 h later. Dorsal hypothalamic regions including the PVN were dissected. Samples were fixed in 1% formaldehyde solution and sonicated. Control and T_3_ treated samples were used for ChIP with anti-LXR or anti-RXR antibodies or without antibody (Ab^-^, negative control). Precipitated DNA fragments were purified. Primers spanning the most conserved nTRE identified in *Trh* (site 4) promoter were used in qPCR to measure enrichment of DNA samples. Negative controls comprised primers spanning irrelevant sequences in gene sequence. Detailed ChIP protocol is provided in SI of [12].

### QPCR

Hypothalami of pups were dissected. Total RNA was extracted. Concentration of total RNA was measured, and RNAs were stored in Tris 10 mM/EDTA 0.1 mM (pH 7.4) at −80°C. To quantify mRNAs in hypothalami, 1 µg of total RNA was reverse-transcribed using the High Capacity cDNA Reverse Transcription kit (Applied Biosystems). Selected LXR target genes were: Stearoyl-CoA desaturase 1 (*Scd1*), Peroxisome proliferator-activated receptor α (*Pparα*), ATP-binding cassette (ABC) transporter (*Abcg1*), Lipoprotein lipase (*Lpl*), Sterol regulatory element binding transcription factor 1 (*Srebp1*), Liver X receptors (*Lxrα and Lxrβ*), ATP-binding cassette sub-family A (*Abca1*), Peroxisome proliferator-activated receptor γ (*Pparγ*) and *Mlxipl* (*Chrebp*, Carbohydrate response element binding protein). We tested also the Brain-derived neurotrophic factor (*Bdnf*) that could have a link with LXR signalling, and genes related to inflammation: Tumor necrosis factor-alpha (*Tnfα*), Interleukin-1 alpha (*Il-1α*), Interleukin 6 (*Il-6*), Cyclooxygenase-2 (*Cox-2*) and v-Rel avian reticuloendotheliosis viral oncogene homolog A (*RELA*, also known as *Nf-κb 3* or *p65*). Primers and taqman probes for the detection of LXR target genes and control assays (Mm01197142_m1 for *Scd1*, Mm00440939_m1 for *Pparα*, Mm00437390-m1 for *Abcg1*, Mm00434770_m1 for *Lpl*, Mm01334042_m1 for *Bdnf*, Mm00550338_m1 for *Srebp,* Mm00437262_m1 for *Lxrα*, Mm00443454_m1 for *Lxrβ*, Mm00442646_m1 for *Abca1*, Mm00440945_m1 for *Pparγ*, Mm00498811 for *Chrebp*, (Mm00443258_m1 for *Tnfα*, Mm00439620_m1 for *Il1α*, Mm00446190_m1 for *Il6,* Mm03294838_g1 for *Cox2*, Mm00501346_m1 for *Rela* and Mm99999915_g1 for *Gapdh*) were purchased from Applied Biosystems (Courtaboeuf, France). Direct detection of the PCR products was monitored by measuring the increase in fluorescence generated by the TaqMan probes. Samples containing 2 µL of cDNA, 1 µL of specific probe, and 10 µL of 2×TaqMan universal PCR Master Mix (Applied Biosystems) were prepared in a final volume of 20 µL. The gene-specific PCR products were measured continuously using ABI PRISM 7300 Sequence Detection System (Applied Biosystems) during 40 cycles. All experiments were run using the same thermal cycling parameters (one cycle at 95°C for 10 min and 40 cycles at 95°C for 15 s and 60°C for 1 min). Non-template controls were used to detect non-specific amplification. The threshold cycle (CT) of each target product was determined and kept constant for all data analysis, and ΔCT between target and endogenous control (*Gapdh*) was calculated. The difference in ΔCT values of two groups (ΔΔCT) was used to calculate the fold increase (F = 2^−ΔΔCT^) and to determine the changes in target gene expression between control and treated group.

### Statistical Analysis

For *in vivo* gene transfer, non-parametric permutation test (Cytel Studio software) was used to assess statistical significance. Each experiment was carried out with n≥10, repeated at least three times providing the same results. Differences were considered significant at p<0.05 with *, p<0.05; **, p<0.01; ***, p<0.001. QPCR results: Statistical analysis for qPCR data compared ΔΔCT values using non-parametric ANOVA, followed by a permutation test to compare control and treated groups. Independent experiments (7≤n≤8) were repeated two times providing similar results and data were pooled.

## Results

### GW3965 represses the transcription from *Trh* and *Mc4r* promoters only in euthyroid newborn mice

**Figure 1:**
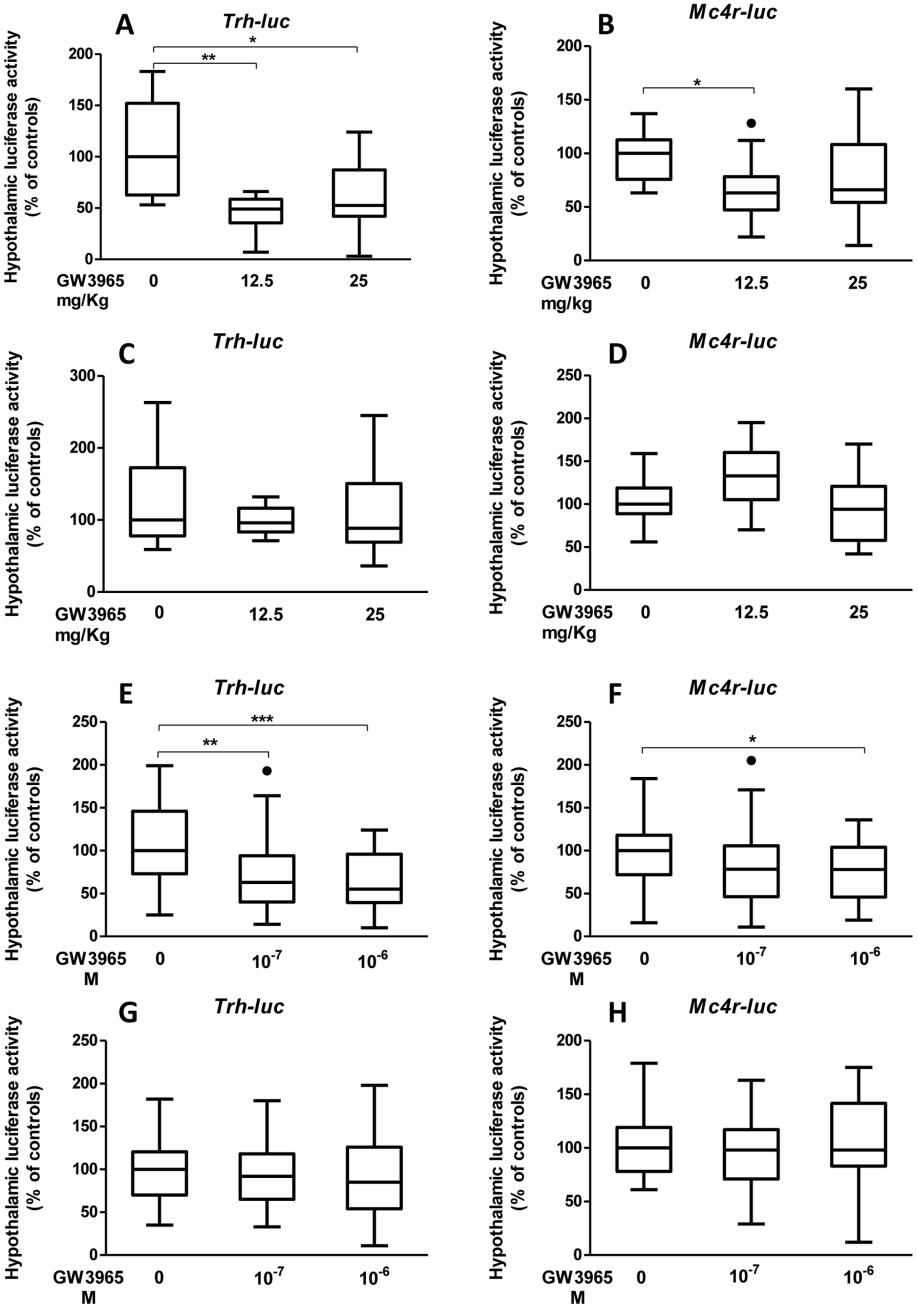
GW3965 represses hypothalamic *Trh* and *Mc4r* transcriptional activity only in euthyroid newborn mice. **A** and **B**. Transcriptional repression of *Trh* (A) and *Mc4r* (B) promoters via LXR activation by subcutaneous injection of GW3965 (12.5 or 25 mg/Kg) in euthyroid newborn mice. **C** and **D**. Lack of effect of subcutaneous injection of GW3965 (12.5 or 25 mg/Kg) on the transcription from *Trh* (C) and *Mc4r* (D) promoters in hypothyroid newborn mice. **A–B/C–D**. *In vivo* gene reporter assays: one-day-old euthyroid (A and B) or hypothyroid (C and D) pups were subcutaneously injected by GW3965 at 12.5 or 25 mg/Kg of body weight, and co-transfected 24 h after in the hypothalamic region of the brain (Paraventricular nuclei, PVN) with 4 µl of a solution of PEI–complexed TRH-f.luc (0.2 µg/pup)/MC4R-r.luc (0.8 µg/pup). Firefly and Renilla luciferase activities were measured 24 h later. Representative experiments are shown. n = 10 per group. Non-parametric permutation test was used to assess statistical significance. *, p<0.05, **, p<0.01. **E** and **F**. Transcriptional repression of *Trh* (E) and *Mc4r* (F) promoters via LXR activation by ICV injection of GW3965 (10^−6^ or 10^−7^ M) in euthyroid newborn mice. **G** and **H**. Lack of effect of I.C.V injection of GW3965 (10^−6^ or 10^−7^ M) on the *Trh* (G) and *Mc4r* (H) transcriptions in hypothyroid newborn mice. **E–F/G–H.** One-day-old euthyroid (E and F) or hypothyroid (G and H) pups were co-transfected in the hypothalamic region of the brain (PVN) with a solution of PEI–complexed TRH-f.luc (0.2 µg/pup)/MC4R-r.luc (0.8 µg/pup) with GW3965 at 10^−7^ or 10^−6^ M in the transfection mix. Firefly and Renilla Luciferase activities were measured 24 h later. n = 10 per group, pools of three independent experiments are represented. Non-parametric permutation test was used to assess statistical significance. *, p<0.05, **, p<0.01; ***, p<0.001.

We analysed by *in vivo* gene transfer the effects of a synthetic LXR ligand, GW3965, on *Trh* and *Mc4r* transcriptional activity by subcutaneous or intracerebroventricular injection of newborn mice. One-day-old euthyroid or hypothyroid pups were s.c. injected by GW3965 at 12.5 mg/Kg or 25 mg/Kg of body weight and were co-transfected 24 h after in the hypothalamic region with TRH-f.luc (0.2 µg/pup)/MC4R-r.luc (0.8 µg/pup) as described in material and methods section. Hypothalami were recovered 24 h later and Firefly and Renilla luciferase activities were measured. We showed by *in vivo* gene transfer of TRH- and MC4R-luciferase reporter plasmids in absence or presence of GW3965 that activation of LXR by its synthetic ligand GW3965 induces repression of the transcriptional activity of *Trh* (Fig. 1A, p = 0.0017 and p = 0.0257) and *Mc4r* (Fig. 1B, p: 0.0255) promoters in the hypothalamus of euthyroid mice. Interestingly, this activation does not occur in hypothyroid mice (Fig. 1C and D). In complementary experiment, one-day-old euthyroid or hypothyroid pups were transfected in the hypothalamic region of the brain (PVN) with 4 µl of a solution of a transfection mix containing PEI-complexed TRH-f.luc (0.2 µg/pup)/MC4R-r.luc (0.8 µg/pup) and GW3965 at 10^−7^ or 10^−6^ M. Hypothalami were dissected 24 h later and Firefly and Renilla luciferase activities were measured. With the second mode of delivery, we obtained the same result as s.c. treatment, *i.e.* activation of LXR by GW3965 inducing repression of the transcriptional activity of *Trh* (Fig. 1E, p: 0.0016 and p: 0.0001) and *Mc4r* (Fig. 1F, p: 0.0268) promoters in the hypothalamus of euthyroid mice, confirming a dialogue between central signalling pathways of TR and LXR. As for s.c. treatment this activation does not occur in hypothyroid mice (Fig. 1G and H) showing the role of T_3_ in this dialogue. So, we studied the effect of thyroid status on TR/LXR dialogue.

### Thyroid hormone treatment of hypothyroid newborn mice or dams restores the GW3965-dependent repression from Trh promoter, but not that from Mc4r promoter in PVN

**Figure 2:**
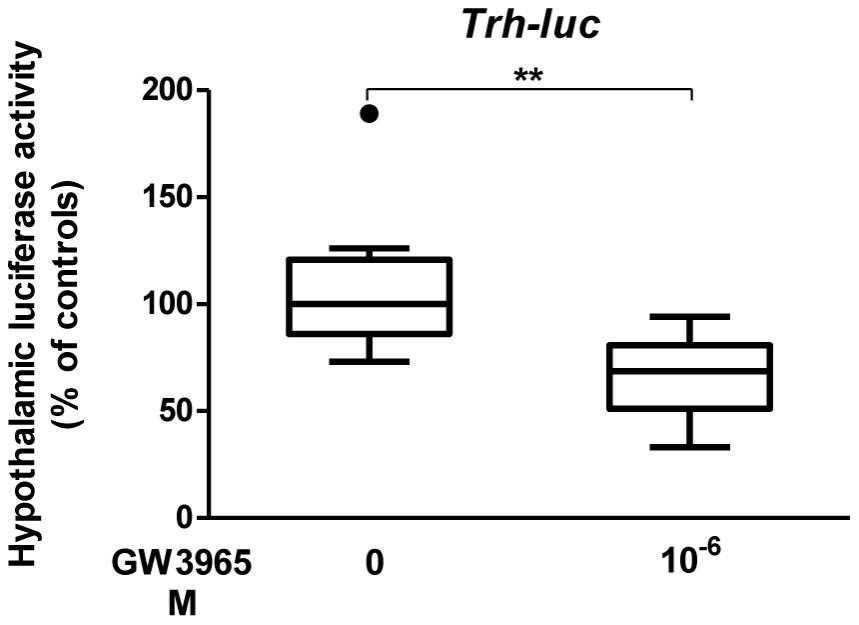
Subcutaneous T_3_ treatment of hypothyroid newborn mice restores the GW3965-dependent repression of the *Trh* promoter. One-day-old hypothyroid pups were subcutaneously injected by T_3_ (2.5 µg/g bw) and co-transfected 24 h after in the hypothalamic region of the brain (Paraventricular nuclei, PVN) with a solution of PEI-complexed TRH-f.luc (0.2 µg/pup)/MC4R-r.luc (0.8 µg/pup) with GW3965 at 10^−6^ M in the transfection mix. Firefly and Renilla luciferase activities were measured 24 h later. n = 10 pups per group. A representative experiment is shown. Non-parametric permutation test was used to assess statistical significance, **, p<0.01.

**Figure 3:**
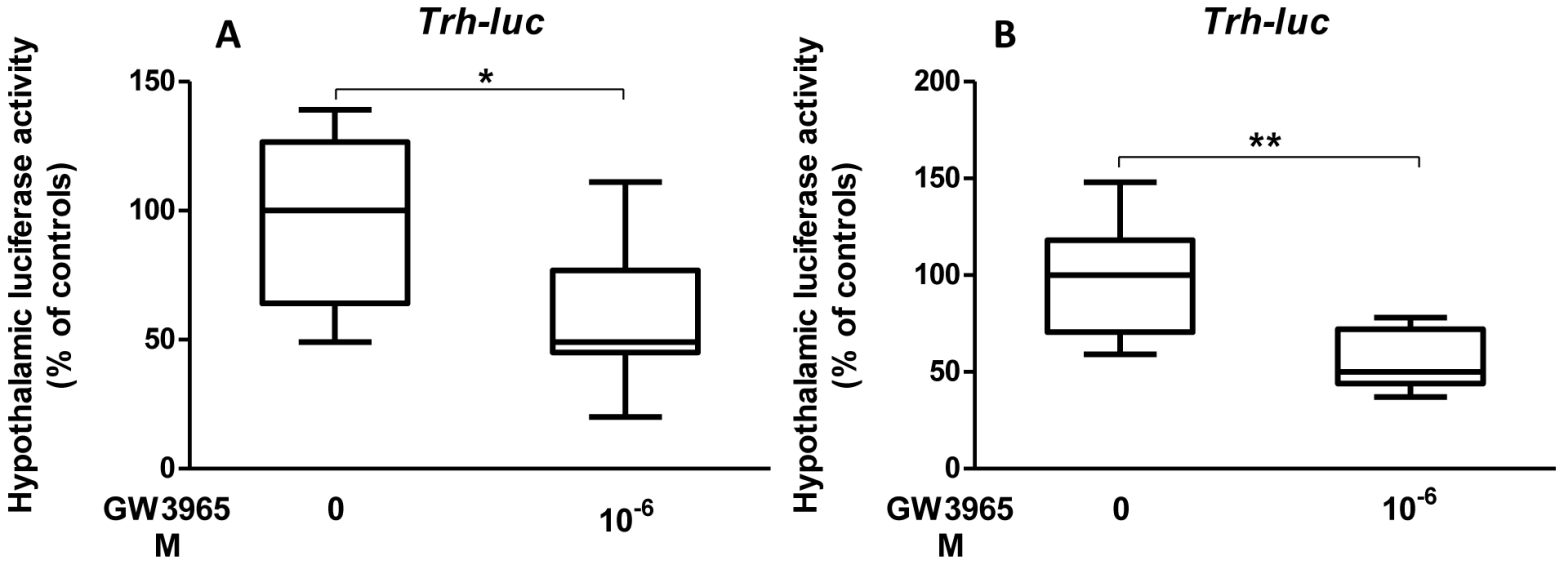
T_4_ treatment of hypothyroid dams restores the GW3965-dependent repression of *Trh* promoter of newborn offspring. **A.** One-day-old hypothyroid pups, from dams treated with T_4_ at 12 µg/ml in drinking water 24 h before giving birth, were transfected in the hypothalamus with a solution of PEI-complexed TRH-f.luc (0.2 µg/pup)/MC4R-r.luc (0.8 µg/pup) with GW3965 at 10^−6^ M in the transfection mix. Firefly and Renilla luciferase activities were measured 24 h later. A representative experiment is shown. n = 10 per group. Non-parametric Mann-Whitney test was used to assess statistical significance. *, p<0.05. **B.** One-day-old hypothyroid pups, from dams treated with T_4_ at 12 µg/ml in drinking water 48 h before giving birth, were co-transfected in the hypothalamus with a solution of PEI-complexed TRH-f.luc (0.2 µg/pup) MC4R-r.luc (0.8 µg/pup) with GW3965 at 10^−6^ M in the transfection mix. Firefly and Renilla luciferase activities were measured 24 h later. A representative experiment is shown. n = 10 per group. Non-parametric permutation test was used to assess statistical significance. **, p<0.01.

To determine whether thyroid hormone plays a role in the LXR/TR dialogue and if its presence is able to restore the transcriptional regulation of *Trh* or *Mc4r* by LXR in hypothyroid newborn mice hypothalami, one-day-old hypothyroid pups were s.c. injected by T_3_ (2.5 µg/g bw) 24 h before they were co- transfected by TRH-f.luc (0.2 µg/pup)/MC4R-r.luc (0.8 µg/pup) mix as in the previous experiment. Hypothalami were dissected 24 h later and Firefly and Renilla Luciferases measured. Subcutaneous triiodothyronine treatment of hypothyroid newborn mice restored in the hypothalamus the GW3965-dependent repression of the *Trh* promoter (p: 0.0025) (Fig. 2) but not that of *Mc4r* promoter (data not shown). For the same purpose, we treated dams with T_4_ at 12 µg/ml in the drinking water for 24 h before giving birth, and pups (d1) were co-transfected in the hypothalamus by TRH-f.luc (0.2 µg/pup)/MC4R-r.luc (0.8 µg/pup) simultaneously with GW3965 at 10^−6^ M. Hypothalami were recovered 24 h later and Firefly and Renilla luciferases activities measured. As shown in Fig. 3A (representative experiment), T_4_ treatment of hypothyroid dams restored the ligand-dependent repression of *Trh* (p: 0.0145) but not of MC4R (data not shown). These effects were also observed when the T_4_ treatment began 48 h before birth (Fig. 3B, p: 0.0094).

### The repression of hypothalamic Trh activity by LXR agonist in euthyroid newborn mice is dependent on the presence of LXR

**Figure 4:**
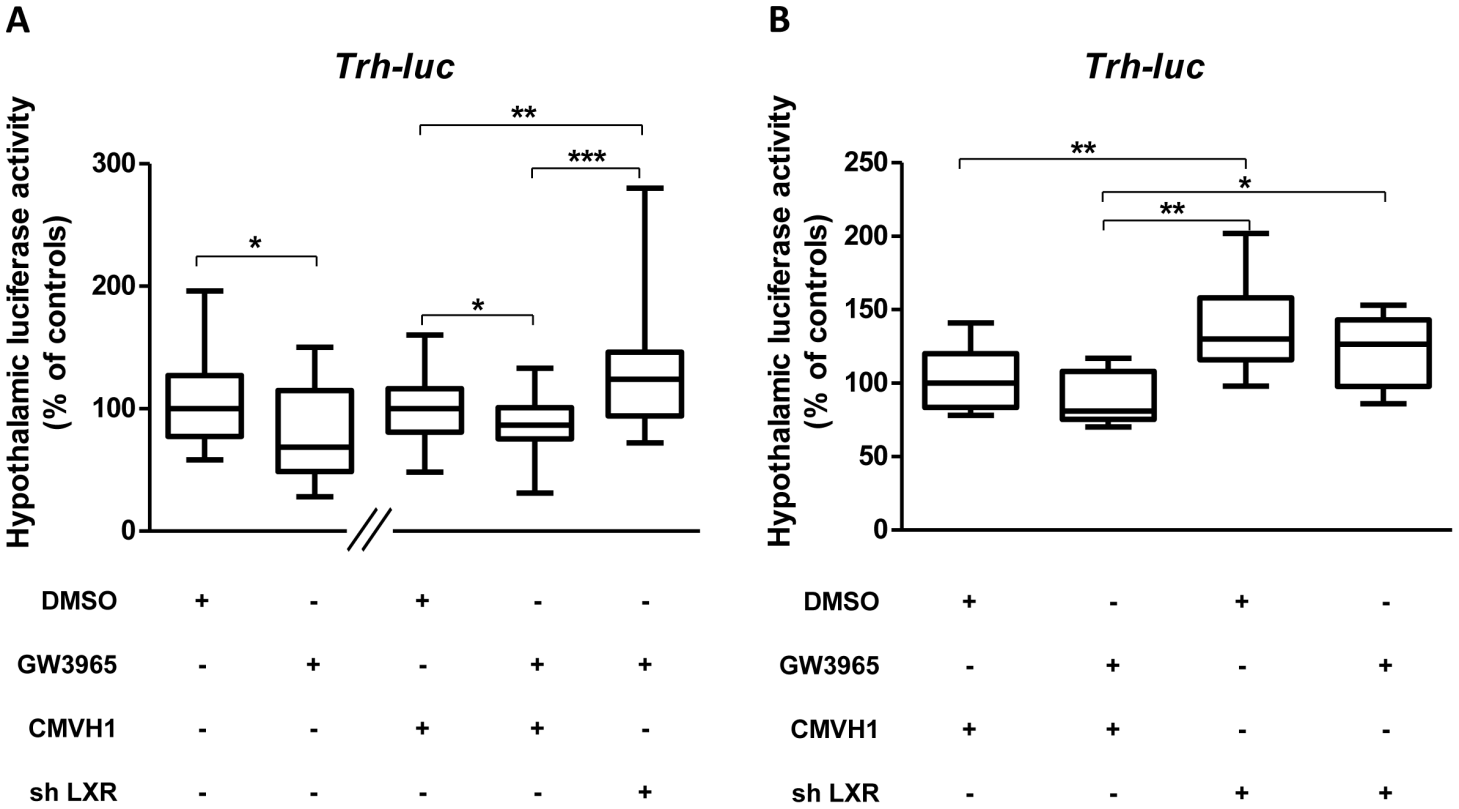
The repression of hypothalamic *Trh* activity by LXR agonist in euthyroid newborn mice is dependent on the presence of LXR. A: LXRs are obligatory for transcriptional repression of *Trh* promoter by GW3965 (10^−6^ M) in euthyroid newborn mice. B: Transcriptional activation of *Trh* promoter when LXR is knocked-down by ICV injection of shLXR in euthyroid newborn mice. One-day-old euthyroid pups were transfected in the hypothalamic region of the brain (PVN) with 4 µl of a solution of a transfection mix containing PEI-complexed TRH-f.luc (0.2 µg/pup) alone (first two columns in A) or with empty CMV-H1 (0.4 µg/pup) (following two columns in A, first two columns in B) or a mix of CMV-H1shLXRα (0.2 µg/pup) and CMV-H1shLXRβ (0.2 µg/pup) (last column in A, and last two columns in B) with (+) or without (−) GW3965 at 10^−6^ M. Firefly Luciferase activity was measured 24 h later. n = 10 or 12 per group, A: pools of two (first part of the graph) or three (second part of the graph) independent experiments and B: one representative experiment. Non-parametric permutation test was used to assess statistical significance. *, p<0.05, **, p<0.01; ***, p<0.001.

To further analyse the implication of LXR in the observed repression of *Trh* transcriptional activity by LXR synthetic ligand GW3965, we compared by *in vivo* gene transfer the effects of GW3965 on *Trh* transcriptional activity in presence or absence of LXRs by cotransfecting newborn mice with TRH-f. Luc and a mix of plasmids expressing shLXRα and shLXRβ. One-day-old euthyroid pups were transfected in the hypothalamic region of the brain (PVN) with 4 µl of a transfection mix containing PEI-complexed TRH-f.luc (0.2 µg/pup)/CMV-H1-shLXRα (0.2 µg/pup) and CMV-H1-shLXRβ (0.2 µg/pup) (or 0.4 µg/pup of empty CMV-H1) with or without GW3965 at 10^−6^ M. Hypothalami were dissected 24 h later and Firefly luciferase activity was measured. We confirmed the result obtained in the first experiment i.e. GW3965 inducing repression of the transcriptional activity of *Trh* promoter (Fig. 4A second and fourth columns; p: 0.04401 (without CMV-H1, second column) and 0.02952 (in presence of CMV-H1, fourth column)) in the hypothalamus of euthyroid mice and this before knocking-down LXR. By adding shLXRα-β in the transfection mix, we observed an activation of the transcriptional activity of *Trh* promoter (Fig. 4A, p:0.00174 and p:1e-005 compared respectively to without (third column) or with (fourth column) GW3965). Interestingly, this activation of TRH-Luc by knocking-down LXRα-β was observable even in the absence of GW3965 (Fig. 4B, third column compared to second p: 0.0073).

### LXR and RXR are not detected simultaneously on Trh promoter

**Figure 5:**
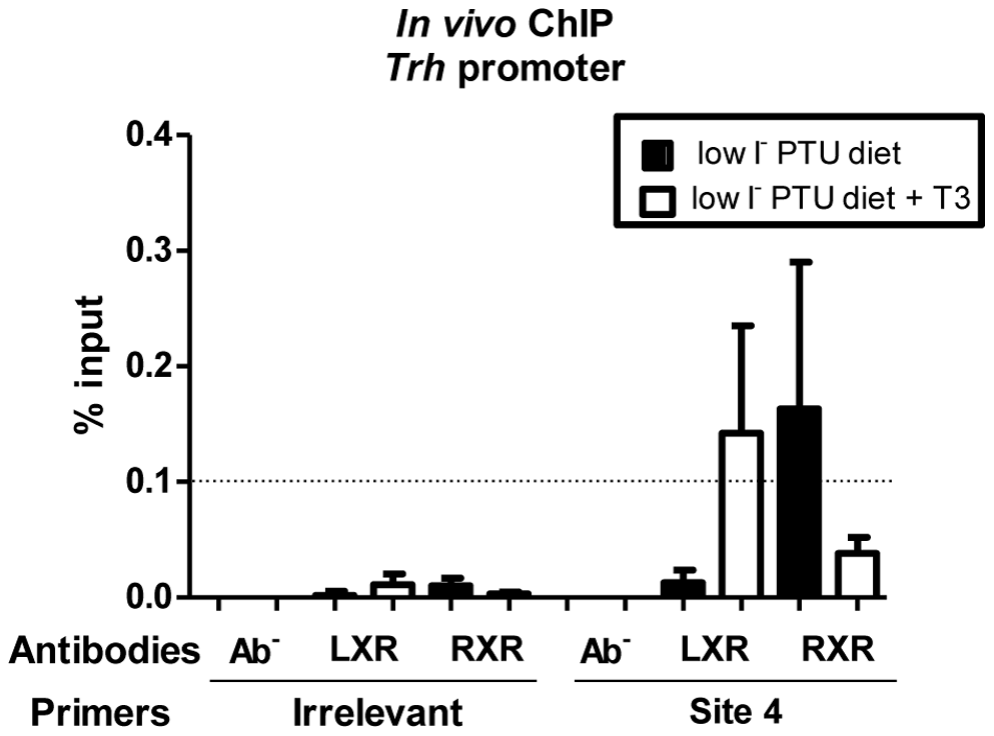
LXR and RXR are not detected simultaneously on *Trh* promoter. PCR quantification of Chromatin Immunoprecipitation (ChIP) assays carried out on hypothalami from hypothyroid newborn mice treated or not with T_3_ (2.5 µg/g bw) 20 h before sacrifice. Samples were immunoprecipitated with LXR- or RXR- specific antibodies and amplified with TRH site 4 primer or its irrelevant control primer (TRH -2000). For negative controls, samples were processed through immunoprecipitation without antibody (Ab^−^). Results represent the occupancy of LXR and RXR at the TRE-site 4 in *Trh* promoter. Data are presented as percentage of input (starting sonicated DNA used for ChIP). The threshold value for a positive signal was set at 0.1% of input (dashed-line). The results are presented as percentage of input of (Ab+) minus (Ab–). The graph represents means of three independent experiments. LXR isoform is present at the TRE site 4 in the *Trh* promoter in hypothyroid animals only after T_3_ treatment. RXR isoform is present at the TRE site 4 in the *Trh* promoter in hypothyroid animals but absent after T_3_ treatment. There is no significant simultaneous recruitment of LXR and RXR to the site 4 of *Trh*.

In order to uncover the transcriptional mechanism underlying the regulations described above, we analysed the occupancy of *Trh* promoter by LXR and RXR by using the technique of *in vivo* ChIP. LXR or RXR binding was analysed on the most conserved nTRE identified in *Trh* (site 4) regulatory region. ChIP using anti-LXR or anti-RXR antibodies was carried out on hypothalami from hypothyroid newborn mice treated or not with T_3_ for 20 h. Results represent the occupancy of LXR and RXR at the TRE-site 4 in *Trh* promoter (Fig. 5). These experiments showed that LXR is recruited to the *Trh* promoter region in the presence of T_3_ (Fig. 5, third column from the right) but not in its absence (Fig. 5, fourth column from the right). In contrast, RXR is recruited to the same region in the absence of T_3_ (Fig. 5, second column from the right) as already described [18]. We also note that there is no significant simultaneous recruitment of LXR and RXR to *Trh* promoter, suggesting that the presence of one NR excludes the other.

### Some key LXR target genes involved in metabolism and inflammation are regulated by thyroid status in the hypothalamic PVN of newborn mice

**Figure 6:**
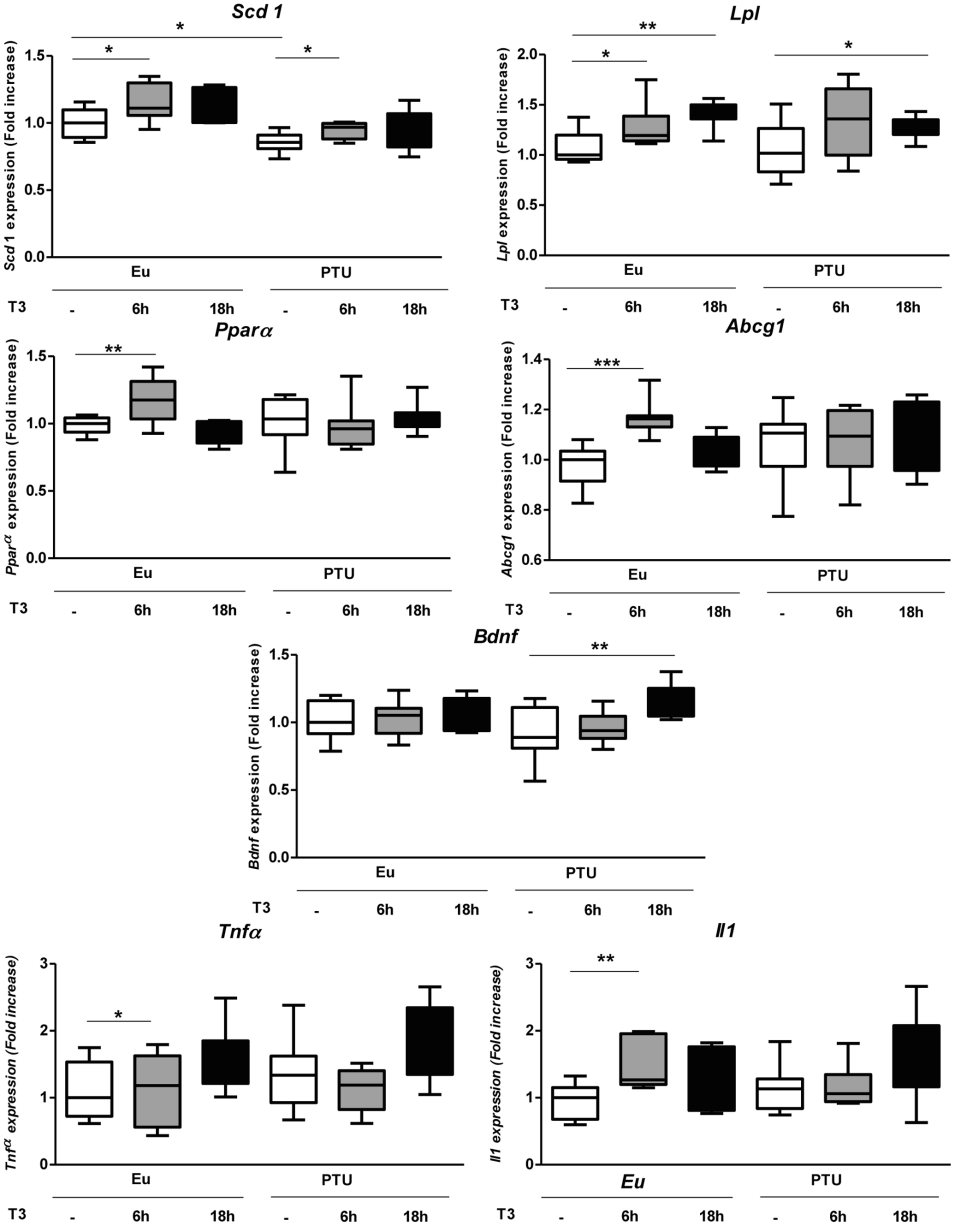
T_3_ regulation of LXR target genes involved in metabolism in the PVN of newborn mice. Eu (controls) - and hypothyroid (from dams treated by PTU the last week of gestation) newborn mice are subcutaneously treated by T_3_ (2.5 µg/g bw) or vehicle 6 h and 18 h before dissection. Hypothalami (PVN) were recovered and PCR quantification of some LXR target gene mRNA from these PVN was done. Gene expression was normalized with *Gapdh*. n = 8 per group, a pool of two independent experiments is shown. Non-parametric ANOVA followed by permutation test with strata was used to assess statistical significance. *, p<0.05, **, p<0.01; ***, p<0.001.

LXR activation represses the transcriptional activity of T*rh* and *Mc4r* promoters, confirming a crosstalk between TR and LXR and thus, regulates genes undergoing feedback by T_3_ in the hypothalamic PVN and involved in metabolism. TH in the periphery regulates several LXR target genes. Thus, we hypothesized that T_3_ could also regulate some LXR target genes within the hypothalamus. To test this possibility, eu- vs hypothyroid newborn mice (day 1) were either treated with T_3_ (2,5 µg/g bw) or vehicle 6 or 18 hours before dissection. Hypothalami (PVN) were dissected and effects of thyroid status on some LXR target gene mRNA expression were quantified by qPCR (Fig. 6). T_3_ treatment of euthyroid pups significantly increased *Abcg1* (6 h after T_3_ treatment, p<0.001, p: 0.00035), *Lpl* (6 h after T_3_ treatment, p<0.05, p: 0.0339; 18 h after T_3_ treatment, p<0.01, p: 0.0014), *Pparα* (6 h after T_3_ treatment, p<0.01, p: 0.00684) and *Scd1* (6 h after T_3_ treatment, p<0.05, p: 0.02102) mRNA levels, *versus* euthyroid controls (Eu, Fig. 6). However, hypothyroidism modified only *Scd* (Eu –T_3_ vs PTU –T_3_, p = 0.01082<0.05) expression in the PVN. As for hypothyroid pups (PTU, Fig. 6), T_3_ treatment increased *Bdnf* (18 h after T_3_ treatment, p<0.01, p: 0.009), *Lpl* (18 h after T_3_ treatment, p<0.05, p: 0.02789) and *Scd* (6 h after T_3_ treatment, p<0.05, p: 0.02285) mRNA levels.

We looked at other genes involved in cholesterol homeostasis (*Srebp*, *Lxrα* and *Lxrβ*) or encoding the ATP-binding cassette transporters (*Abca1*) as well as genes implicated in lipid metabolism (*Pparγ* and *Chrebp*) and we noticed no significant effect of T_3_ on mRNA expression levels (data not shown).

Regarding expression of LXR target genes involved in inflammation, T_3_ treatment of euthyroid pups significantly increased *Tnfα* (18 h after T_3_ treatment, p<0.05, p: 0.01643) and *Il1* (6 h after T_3_ treatment, p<0.001, p: 0.0049) mRNA levels (Fig. 6). Hypothyroidism and T_3_ treatment of hypothyroid newborn mice had no significant effect on the expression of *Tnfα* and *Il1* mRNA levels. The other genes studied (*Il6*, *Nfκb* and *Cox2*) were not significantly affected by either treatment (data not shown).

## Discussion

Both TRs and LXRs play important roles in metabolic regulation [19]–[22]. Crosstalks between pathways regulated by these two NRs have been reported, especially regarding lipid metabolism-related genes [8] but also in other physiological systems such as the central nervous system [23] as LXRs are expressed in, notably, the brain stem, the hypothalamus and the cortex [24]. 24-(S)-hydroxycholesterol-liganded LXR regulates cholesterol availability in the brain playing an important role in hypothalamic cholesterol homeostasis, which is crucial for brain physiology [25]. In addition, the study of Perez-Tilve et al. [14] shows a role for the melanocortin system in controlling hepatic cholesterol metabolism. Inhibition of the melanocortin system increases circulating HDL cholesterol levels by reducing its uptake by the liver. Thus, there could be a crosstalk between pathways governed by LXR and TR in the hypothalamus, in the context of maintenance of lipid homeostasis.

Thus, our objective was a greater understanding of the physiological links between TR and LXR signalling systems, in the context of central control of energy homeostasis. In particular, our hypothesis was that LXR might modulate TR-dependent *Trh* and *Mc4r* transcriptions. This was verified by *in vivo* gene transfer and shRNA strategy to knock-down LXRs. Our data show that hypothalamic LXR, when activated by its synthetic ligand, has a repressive action on *Trh* and *Mc4r* promoters. Interestingly, when both LXRs where knocked-down, the repression on *Trh* promoter transcriptional activity was lost (with or without the ligand GW3965), resulting in an activation of transcription from the promoter compared to controls (with no GW3965, but with LXRs, fig. 4B). This result indicates that LXR is able to repress *Trh* promoter activity even when it is not activated by synthetic ligand. Thus, endogenous LXR ligand could activate LXR to repress *Trh* promoter. When LXRs are knocked-down, this repression is released, leading to a relative activation of the *Trh* promoter. This result can be linked with previous results showing LXR to repress *Dio2* transcription [26]. In both cases, the repressive effect of LXR would lead to a decrease in T_3_ production, thus showing a global inhibitory effect of LXR on T_3_ driven pathways.

It is interesting to note that the repressive action of LXR on *Trh* and *Mc4r* promoters is only observed in euthyroid newborn mice. Thus, LXR-dependent regulation of *Trh* and *Mc4r* transcription seems to be influenced by thyroid status in the PVN. Thyroid status is also known to specifically regulating *Mc4r* expression in the PVN, with hypothyroidism increasing endogenous *Mc4r* expression [12], which parallels the well-established rise in PVN *Trh* expression also induced by hypothyroidism [27], [28]. We further explored the importance of the thyroid status by studying the concomitant effect of T_3_ and GW3965 on TR/LXR dialogue. TH replacement in hypothyroid hypothalamus restored the GW3965-dependent repression of the *Trh* promoter by LXR but not that of *Mc4r.* These different responses could be explained by a difference in the mechanisms involved, in particular, regarding TR-induced regulations. Data from many *in vitro* studies on positively regulated genes suggest a model wherein TRs bind to pTREs with or without its ligand, T_3_ [29]. *In vivo*, the nTREs studied by Decherf *et al.* in the *Mc4r* and *Trh* promoters showed distinct TRβ recruitment patterns as a function of T_3_ presence [12]. *Mc4r* TRE1 recruited only low levels of TRβ in the absence of T_3_, whereas T_3_ induced a large increase of TRβ binding. In contrast, TRβ was found on *Trh* TRE site 4 without hormone, as previously shown [30], and T_3_ induced the dissociation of TRβ from *Trh* TRE site 4 (see Fig. 2B and Supporting Information, Fig. S4 in [12]). These differences may contribute to the differences observed on LXR mediated regulations in addition to the different sensitivities to T_3_-dependent repression of these two genes. In the present study, the *in vivo* ChIP experiments showed that LXR is recruited to the *Trh* promoter region in the presence of T_3_ but not in its absence. In contrast, RXR is recruited to the same region in the absence of T_3_ (as found by Decherf et al [18]). We also note that there is no significant simultaneous recruitment of LXR and RXR to the site 4 of *Trh* promoter, suggesting that the presence of a NR excludes the other. However, in periphery, LXRs generally function as permissive heterodimer with RXR. But it is worth to note that this heterodimer is observed on LXRE, which is a DR4 (that means, two half-sites present for binding of NR) whereas we studied LXR binding on the most conserved nTRE identified in *Trh*, the site 4, a half-site, which could explain the absence of RXR. Furthermore, the *Trh* site 4 preferentially binds TR/RXR heterodimers [31]. Consequently, we could propose a model where, in hypothyroid mice, TRβ would be recruited to the *Trh* site 4 as a heterodimer with RXR to activate the ligand–independent transcription. After T_3_ treatment, TRβ could dissociate from the site 4 where LXR would be then recruited and represses *Trh* transcription. It has been reported [12] for *Mc4r,* that in a hypothyroid state, TRE1 recruited low levels of TRβ but T_3_ treatment induced a large increase of TRβ binding. The recruitment of LXR could consequently be inhibited and then, prevent the regulation of *Mc4r* transcription by LXR, observed in a euthyroid state. These data provide a basis for a model for LXR interference with *Trh* and *Mc4r* transcription.

Thus, the data show that LXR represses the transcription of T_3_ regulated genes involved in central control of metabolism in the hypothalamic PVN. To further test the hypothesis that T_3_ could also regulate key LXR target genes in the hypothalamus we used qPCR to analyse potential central TR/LXR crosstalk.

In the periphery, LXRs regulate a variety of genes encoding diverse enzymes (for review see, [5], [8]) involved in cholesterol metabolism such as ABCG1 and Cyp7A1 [32]–[34] as well as in hepatic lipogenesis (e.g. FAS and SCD1) [35], [36] and in lipoproteins metabolism (LPL, CETP, etc) [37]. Many of these LXR target genes are also regulated by TH in the periphery [8], [38]–[40] and then could be regulated by T_3_ in the hypothalamus. We selected some of these LXR target genes to investigate potential central T_3_ regulation, which could in turn be linked to central control of metabolism. As for *in vivo* gene transfer experiments, where we only saw effects of LXR ligand in euthyroid animals, again in qPCR analysis of T_3_ regulation of LXR target genes expression, we observed more regulations in the euthyroid than in the hypothyroid group, emphasising the importance of the thyroid status in the crosstalk between LXR and TR signalling. Our data show that T_3_ significantly increased *Scd-1* mRNA levels in PVN from euthyroid and hypothyroid pups. Moreover, *Scd-1* mRNA level was lower in hypothyroid than in euthyroid pups. Thus, *Scd-1* is positively regulated by TH in the PVN region of newborn mice. SCD-1 is one of the major enzymes involved in fatty acid metabolism and converts saturated fatty acids into monounsaturated fatty acids [41]–[43]. At the central level, a possible link between SCD-1 and T_3_ could be related to anti-inflammatory action and control of food intake. Indeed, hypothalamic inflammation is an early signal of the onset of obesity, due to abnormal control of caloric intake (review in [44]). Moreover, Cintra and colleagues [45] show that linolenic and oleic fatty acids (unsaturated fatty acids) inhibit the AMPK/ACC pathway (as does T_3_) [46] while increasing SCD1 expression in the hypothalamus, again, as does T_3_ in our study. Furthermore, fatty acids injections significantly reduce spontaneous food intake and enhance the anorexigenic effect of leptin [45].

Leptin is also well known to regulate TRH, either directly [47], or indirectly, through MC4R activation [48], and both TH and leptin signalling activation induce anorexigenic pathways. *Lpl* was also regulated by T_3_ in the newborn mice PVN. LPL is a multifunctional enzyme that plays a major role in the metabolism and transport of lipids in peripheral tissues [49], [50]. In the brain, LPL is important to energy balance and body weight regulation, influencing the same pathways as SCD, *i.e* unsaturated fatty acid control of food intake. In particular, neuronal LPL deficiency leads to hypothalamic AgRP up-regulation (review in [51]), thereby activating orexigenic pathways. Hypothetically, LPL would provide important lipid-derived regulatory signals such as polyunsaturated fatty acids (PUFAs), which in turn regulate AgRP expression in the hypothalamus and thus energy balance and body weight. However, AgRP signalling inhibits HPT axis (review in [11]) and T_3_ up-regulates *Lpl* expression. Thus, these results confirm that AgRP and T_3_ act in an antagonistic manner, the former stimulating orexigenic pathways, whilst the latter inhibits them.

Thus, it appears that LXR and TR have common target genes and pathways in the hypothalamus. Taken together our results suggest that LXR and TR crosstalk within the hypothalamus could be involved in the central control of food intake, and more generally, in the regulation of energetic homeostasis.

Another common link between these gene networks could be their implication in neurodegenerative disease and inflammation. In fact, LXRs have emerged as important regulators of the innate and adaptive immune system and inflammation [32]. LXR signalling impacts the development of Alzheimer’s disease (AD) pathology and LXRs are promising therapeutic targets for AD treatment because of their ability to affect components of the disease such as cholesterol content, Aβ clearance, APP processing, ABCA1, etc. [52]. It has been recently demonstrated that TR and LXR competitively up-regulate the human selective AD indicator-1 (Seladin-1) gene promoter at the transcriptional levels and both receptors share a positive TRE/LXRE [23]. Indeed, BDNF, like LXR, is involved in cholesterol metabolism and in neurodegenerative disease [53]. In hypothyroid pups we report that T_3_ treatment increases *Bdnf* mRNA levels. Crupi *et al*. [54] have also reported that T_3_ significantly enhanced the post-traumatic brain injury expression of the neuroprotective neurotrophin BDNF, showing a potential anti-inflammatory effect of T_3_. Further, we observed a significant T_3-_dependent increase in *Pparα* mRNA levels in the hypothalamic region. Thus, *Pparα* is centrally regulated by T_3_ and may be involved in the central regulation of lipid metabolism. Indeed, it is established that, in the periphery, *Pparα* is a LXR/TR target, and extensive data establish the importance of PPARα in inflammation [55]. Thus, *Pparα* may be involved at the central level in inflammation. In addition, synthetic LXR agonists have been shown to have anti-inflammatory features [56]. The anti-inflammatory activities of LXR were described in 2003 [57] using a cutaneous inflammatory mouse model in which activation of LXR by GW3965 or 22-hydroxycholesterol inhibited production of TNFα and IL-1α. Interestingly, it has also been demonstrated that a hyperthyroid state in the rat increases circulating levels of TNF-α by actions exerted at the Kupffer cell level, and this is related to the oxidative stress status established in the liver by T_3_-dependent calorigenesis [58]. Our results show that T_3_ also increases *Tnfα* and *Il1* in the hypothalamic region. Thus, the crosstalk between LXR and TH is also involved in central regulation of inflammation. Indeed, those two pathways could act synergistically to reduce inflammation, LXR inhibiting *Trh* transcription and thus T_3_ secretion, leading to a subsequent reduction of TNF*α* and IL1 production, reinforcing the repressive effect of LXR on these two factors [57].

Thus, LXRs could be attractive drug targets for therapeutic intervention in metabolic disorders and inflammatory diseases even at the central level. RXR also plays an important role in the regulation of inflammation [59]. Finally, TRs act also as inhibitors of inflammation [60]. These data together lead us to suggest that it could exist an interaction between TR/RXR and LXR at the central level to regulate inflammation in addition to the metabolism regulation.

In conclusion, this study represents the first *in vivo* report of a central LXR/TR signalling crosstalk, in a brain region relevant to metabolic homeostasis, the hypothalamus. The dual control of central metabolic and inflammatory pathways could lead to a fine tuning, allowing for synergetic regulations. Moreover, furthering the understanding of the molecular mechanisms of LXR/TR interaction in these regulations could be important for therapeutic intervention.
